# A UPLC-MS/MS Based Rapid, Sensitive, and Non-Enzymatic Methodology for Quantitation of Dietary Isoflavones in Biological Fluids

**DOI:** 10.3390/molecules28186729

**Published:** 2023-09-21

**Authors:** Faraz Rashid, Sudeep Ghimire, Ashutosh K. Mangalam, Shailendra Giri

**Affiliations:** 1 Department of Neurology, Henry Ford Health, Detroit, MI 48202, USA; frashid2@hfhs.org; 2 Department of Pathology, University of Iowa, Iowa City, IA 52242, USA; sudeep-ghimire@uiowa.edu

**Keywords:** dietary isoflavones, non-enzymatic, UPLC-MS/MS

## Abstract

Dietary isoflavones, a type of phytoestrogens, have gained importance owing to their health-promoting benefits. However, the beneficial effects of isoflavones are mediated by smaller metabolites produced with the help of gut bacteria that are known to metabolize these phytoestrogenic compounds into Daidzein and Genistein and biologically active molecules such as S-Equol. Identifying and measuring these phytoestrogens and their metabolites is an important step towards understanding the significance of diet and gut microbiota in human health and diseases. We have overcome the reported difficulties in quantitation of these isoflavones and developed a simplified, sensitive, non-enzymatic, and sulfatases-free extraction methodology. We have subsequently used this method to quantify these metabolites in the urine of mice using UPLC-MS/MS. The extraction and quantitation method was validated for precision, linearity, accuracy, recoveries, limit of detection (LOD), and limit of quantification (LOQ). Linear calibration curves for Daidzein, Genistein, and S-Equol were set up by performing linear regression analysis and checked using the correlation coefficient (r^2^ > 0.995). LOQs for Daidzein, Genistein, and S-Equol were 2, 4, and 2 ng/mL, respectively. This UPLC-MS/MS swift method is suitable for quantifying isoflavones and the microbial-derived metabolite S-Equol in mice urine and is particularly useful for large numbers of samples.

## 1. Introduction

Phytoestrogens are plant compounds which have been linked with health-promoting effects due to their structural similarity to estrogens. Dietary isoflavones are a type of phytoestrogen that are health-promoting components of soybeans. It has been well documented that isoflavones could inhibit cancer cell proliferation [1], enhance antioxidant and anti-inflammation activities [2,3], and prevent osteoporosis [4,5] as well as cardiovascular disease [6], have a role in the improvement of cognitive impairment, antidepressant effects, blood pressure improvements, as well as in renal dysfunction [7,8]. Although the recommended daily intake of isoflavones has not yet been established, the FDA recommends an intake of 10–50 mg per day, which is considered safe [9]. Isoflavone can mediate their effect either directly, through original plant-derived metabolites such as Daidzein and Genistein, or through smaller metabolites such a S-Equol produced with the help of gut bacteria. As S-Equol has a higher binding affinity to estrogen receptors (ERs) specially ERα, it has been suggested that S-Equol may be more beneficial than original phytoestrogens such as Daidzein and Genistein.

The ability of isoflavones to render their beneficial bioactive role depends on absorption, metabolism, distribution to target tissues, and excretion of these compounds, i.e., their bio-accessibility. The absorption and excretion of isoflavones follows a similar route to other lipophilic compounds. Isoflavones exist as glycosides, are absorbed in the intestine and converted to Genistein, Daidzein, Isoformononetin (IFN), and other minor metabolites in the intestine by the gastric acid and the microbial enzyme β-glucosidase [10]. Gut microflora such as *Adlercreutzia S-Equolifaciens*, *Eggerthella* species, *Lactococcus garvieae*, *Slackia S-Equolifaciens*, *Lactococcus garvieae*,and *Senegalimassilia* sp. produce β-glucosidase and sulfatase enzymes in enteric phases (I and II) and hydrolyse the isoflavones before being absorbed through the intestinal brush border system [11]. Ingested Daidzein is further metabolized to dihydrodaidzein, tetrahydrodaidzein, O-desmethylangolensin (O-DMA), and to S-Equol by intestinal bacteria. Being Lipophilic in nature, they join the chylomicron to be transferred via blood along the lymph nodes and be delivered to all cells in the body, and are metabolized via the same pathways as other lipophilic nutrients [12]. When isoflavones reach the liver, they are stored in the form of glucuronide or sulfate, then released to the small intestine through the bile for circulation in the gastrointestinal tract. When the circulation is over, isoflavones are excreted mainly through urine [13].

The isoflavones in human and murine urine can be quantified, as can plasma isoflavones if the isoflavones are ingested in amounts of 10 mg or more per day [14]. Pertinently, it is the S-Equol, an isoflavone-derived metabolite formed from Daidzin/Daidzein by bacteria in the distal region of the small intestine and colon, but not O-DMA, that has estrogenic activity and [15] has been shown to have better affinity for estrogen receptors than Genistein or Daidzein [16]. Therefore, the absorption and metabolism of phytoestrogens demonstrate large interindividual variability, which may relate to differences in both human pharmacokinetics and metabolism by intestinal bacteria. This ability to produce S-Equol may be related to dietary habits and the composition of gut microbiota of an individual, as only 30% of population possesses gut microbiota with the ability to metabolize isoflavones into Equol [17,18]. Due to the greater interest in the beneficial effects of these dietary supplements, their detection and quantification has gained importance.

The separation, identification, and quantification of soy isoflavones are often conducted by high-performance liquid chromatography (HPLC) or gas chromatography (GC) coupled with a mass spectrometry detector [19]. Chromatography-based methods have been employed to detect dietary isoflavones in various matrices. Each method has its advantages and limitations, as well as challenges associated with sample preparation—especially the extraction from a complex matrix—use of different types of sorbent, sample ratio, and the type, volume, and pH of elution solvent including hazardous solvents such as methanol, acetonitrile, diethyl ether, ethyl acetate, dichloromethane, and n-hexane. The latest sensitive methods are also associated with the frequent use of sulfatase mixture, tert-butyl ether, and dimethylformamide (DMF) coupled with enzymatic hydrolysis of their metabolites by a β-glucuronidase [16]. These reagents often produce high background noise and interference with charge competition in mass spectrometry, which leads to low sensitivity and complex data interpretation.

We have developed a new non-enzymatic and sulfatases-free extraction methodology to process biological fluids to reduce the background noise and report a solid-phase extraction protocol for sample clean-up that is simple and hassle-free, with protracted extraction time. We further report the MRM-based UPLC-MS/MS method for quantification of Daidzein, Genistein (isoflavones), and S-Equol (Isoflavandiol) in biological fluids that is ultra-rapid and sensitive.

## 2. Results and Discussion

**Figure 1:**
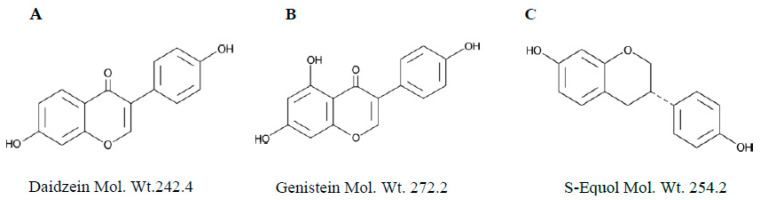
Chemical structures of Daidzein (**A**), Genistein (**B**), and S-Equol (**C**).

The present study was designed to develop a non-enzymatic method for the extraction of dietary isoflavones (Daidzein and Genistein and S-Equol) in biological fluids along with a rapid and sensitive UPLC-MS/MS method developed for quantitation in mice urine samples (Figure 1).

### 2.1. Optimization of Mass Spectrometry and Chromatography Parameters

To achieve maximum efficiency and sensitivity, we optimized the parameters for ionization of Daidzein, Genistein, and S-Equol. Individually standard solutions of Daidzein, Genistein, and S-Equol were infused at the concentration of 1000 ng/mL in 5 mm Ammonium Acetate: Methanol (10:90) at a flow rate of 25 μL/min in a mass analyzer. MS1 mode was activated for the Parent Ion (Q1 ions) scanning, the best ionization parameters (Cone and Extraction voltages) were optimized for highest sensitivity. Daughter (Q3 ions) ions from the same Q1 were selected after fragmentation using optimized collision energy. Electrospray ionization (ESI) with positive and negative ion modes was used to optimize Daidzein, Genistein, and S-Equol to generate the best ionization response, and negative ion mode was found to be the most sensitive with maximum response. Full mass scan spectra of analytes composed of one molecular ion were detected at *m*/*z* 253.2, *m*/*z* 269, and *m*/*z* 241.1 for Daidzein, Genistein, and S-Equol, respectively. A negative Q1 scan for Daidzein (*m*/*z* 253.2) generated two major product ions (M-H) at *m*/*z* 91 and *m*/*z* 223.2. A negative Q1 scan for Genistein (*m*/*z* 269) generated two major product ions of (M-H) at *m*/*z* 133 and *m*/*z* 135. A negative Q1 scan for S-Equol (*m*/*z* 241.1) also generated a two-fragment ion of [M-H] at *m*/*z* 121.2 and *m*/*z* 119.2. These ions were chosen for MRM-based quantification of Daidzein, Genistein, and S-Equol, respectively. Under these experimental conditions, other parameters such as drying gas temperature and flow, nebulizer pressure, and capillary voltage were optimized by flow injection analysis (FIA). Since the UPLC-MS/MS method needs to be optimized to benefit the food industry and academics, we have selected the two best fragmentations for the same Q1 ions as primary and confirmatory ions, as depicted in Table 1 and Table 2. It may also allow for minimal internal standards without compromising selectivity. Unlike conventional methods, this method was developed for its sensitivity and selectivity in the absence of enzyme hydrolysis and glucuronide conjugate. Under these conditions, optimizing the parameters to maintain best sensitivity at low detection levels was one of the challenges for method development. Fine tuning for specific cone voltages, fragmentation energy, and central mass were utilized to overcome these challenges.

### 2.2. Optimization of Chromatography Parameters for Rapid and Sensitive Detection

**Figure 2:**
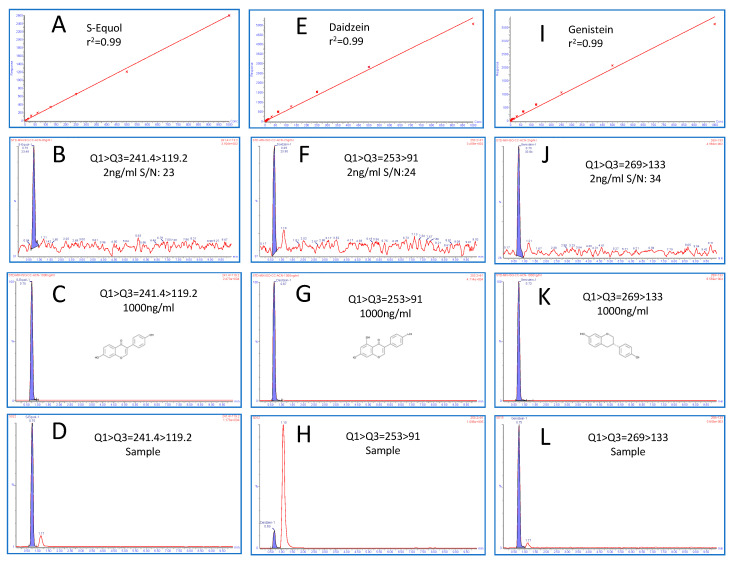
Linear calibration curve (**A**,**E**,**I**), multiple reaction monitoring (MRM) chromatograms for LOD (**B**,**F**,**J**), high-quality control (HQC) chromatogram ((**C**,**G**,**K**) and in vivo mice urine sample (**D**,**H**,**L**)) for S-Equol, Daidzein, and Genistein, respectively.

Numerous reversed-phase UPLC chromatographic separation conditions with different mobile phases and columns were evaluated. We used the following columns: BEH, C18, Atlantis dC18, HILIC, nova pack, and CSH to optimize this method for all three molecules in one single method to maintain the best sensitivity, selectivity, and peak shape, using organic mobile phase buffer (Methanol and Acetonitrile with Formic and acetic acids as additives). Eventually, the sub-2-micron particle UPLC Acquity CSH (C18, 130 Å, 1.7 µm, 2.1 mm × 100 mm) column was found to be the best, with acetic acid as an additive, for ionization and separation. Mobile phase A (2 mM ammonium acetate +0.2% acetic acid) in Aqueous and Mobile phase B (0.2% acetic acid in acetonitrile) at a flow rate of 0.30 mL/min in gradient mode were finalized for the best chromatography and peak shape under UPLC column and temperature conditions at 65 °C. All the three targeted molecules separated very closely with the highest selectivity with the sub-2-micron column for both primary and confirmatory ions. Retention times (min) for Daidzein, Genistein, and S-Equol were 0.69, 0.73, and 0.75, respectively. All steps, including separation, cleaning, and re-equilibration, were completed in a relatively short time (≈3 min). Respective chromatograms for LOD, HQC, and urine samples with the liner calibration curve are depicted in Figure 2.

**Figure 3:**
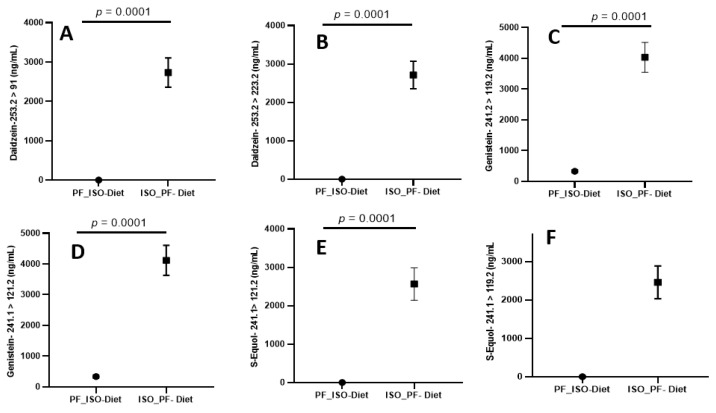
(**A**,**B**) Diadzein, (**C**,**D**) Genistein, and (**E**,**F**) S-Equol quantitation using the rapid LC-MS/MS method in urine samples on phyto-Free (PF) and isoflavone-rich (ISO) diet. Data are expressed as mean ± SD (*n* = 12). Two-tailed unpaired *t*-test. *p* < 0.0001. PF, phyto-Free; ISO, isoflavone-rich.

The newly developed UPLC-MS/MS method was rigorously validated, including the determination of LOD, LOQ, linearity, spiked recovery studies, measures of intra- and inter-day precision, and its application to real urine specimens. Spiked recovery studies were performed to determine the accuracy and precision in urine matrices with anticipated analyte concentrations. The calibration curve was obtained with a pooled urine matrix spiked with a known amount of stock solutions, and also to determine the recovery of a standard for method validation. A nine-point calibration curve (1000, 500, 250, 125, 10, 5, 2, 1, and 0.5 ng/mL) was used to determine linearity, operating linear ranges, and detection limits for all three metabolites. Limits of quantitation (signal to noise > 10) ranged from 2 ng/mL for Daidzein to 4 ng/mL for Genistein and 2 ng/mL for S-Equol. The LOQ to HQC of the calibration range (1–1000 ng/mL) was found to have excellent linearity with regression (r^2^ >0.99) for all the three metabolites for primary and confirmatory ions, as summarized in Table 1 and Table 2. Results indicate that the rapid method developed was highly accurate (91–101.2%) and precise (0.4–9.3% RSD), as shown in Table 3. Method precision was also assessed with intra- and inter-day variations using real urine specimens of C57BL/6J female mice. These tests indicate acceptable intraday variance (1.31–3.6% RSD) and interday variance (3.1–5.0%). Altogether, the results suggest that the newly developed protocol is sufficiently sensitive, selective, and reproducible for analyte quantitation in urine matrices. The robustness of this method to analyze Daidzein, Genistein, and S-Equol in urine samples on phyto-Free (PF) and isoflavone-rich (ISO) diet is validated by the quantitative data acquired, as summarized in Figure 3.

### 2.3. Extraction of Isoflavones from Urine

The extraction of soy isoflavones from urine is often a complex and multi-step process, which involves a variety of solvents such as ethanol, methanol, or acetonitrile, or in combination with water or acid. For nearly two decades, several methods have been reported to improve the extraction of these metabolites from various biological matrices [19]. Most of the published methods need an enzyme and salts, e.g., β-glucuronidase and aryl-sulphatase acid in combination with acid hydrolysis for the extraction [19]. These methods are time-consuming and need extensive clean-up procedures to reduce background interference for mass spectrometry. Keeping in mind the vital need to develop a unique extraction method that can cater to the above sample-related issues, we have optimized and developed a unique extraction method that is non-enzymatic and sulphate-free in nature. Extraction (4 h) and cleanup (2 h) at a specific pH and temperature (65 °C) for all metabolites are described in the Methods Section 3.3. Sample processing time was reduced significantly from 2 days to a single day, compared to most of the commonly published methods [19,20,21,22]. Our method is rapid, robust, and equally sensitive with the advantages of the sub-micron UPLC column where all the three analysts were detected within 2 min. All steps, including separation, cleaning, and re-equilibration, were completed in a relatively short time (≈3 min). High recovery of all three isoflavones ranging from 91 to 101% was attained. The self-explanatory extraction and clean-up procedure is described in the Methods section (Section 3.3).

### 2.4. Method Validation

For identification and quantification of the targeted compounds, we have validated and established the performance characterization of the UPLC-MS/MS-based rapid analytical method with assay in terms of precision, linearity, sensitivity, accuracy, LOD, and LOQ with baseline urine samples. C57BL/6J mice (*n* = 12) were put on a regular diet for 28 days and urine samples were extracted and analyzed for the presence of Daidzein, Genistein, or S-Equol. At the same time, assay spiking was also checked during this intervening period. Urine samples with no detectable isoflavones/S-Equol were selected as baseline controls from mice with a regular diet.

### 2.5. Linear Dynamic Range and Sensitivity

To evaluate the linear relationship of the method, nine calibration standards were subjected to the full extraction procedure three times before analysis. Daidzein, Genistein, and S-Equol reference standards were analyzed over the range of 0.25–1000 ng/mL (Daidzein and S-Equol) and 1–1000 ng/mL (Genistein) in control mice urine to construct calibration curves. The calibration curves were established and fitted with least square regression on a 1/x^2^ weighing linear equation (Table 1). The LODs for Daidzein, Genistein, and S-Equol were 0.5, 1, and 0.25 ng/mL, respectively, while the LOQs were 2, 4, and 2 ng/mL, respectively. Linear calibration curves for Daidzein, Genistein, and S-Equol were set up by performing linear regression analysis and were checked using the correlation coefficient (r^2^ > 0.995). Linear regression analysis was performed by plotting the concentration versus analyte peak area. The LOD and LOQ were defined for all three (Daidzein, Geninstein, and S-Equol) molecules corresponding to the lowest calibration point, where signal-to-noise was more than 3 and 10 times the signal-to-noise ratio (S/N ≥ 3 for LOD and S/N ≥ 10 for LOQ) in the baseline noise of blank samples.

### 2.6. Precision Accuracy and Recovery

Precision and accuracy for this method were evaluated and calculated using matrices spiking with known amounts of Daidzein, Genistein, and S-Equol in urine samples. Each standard was spiked in urine at low 10 ng/mL (*n* = 3), medium 25 ng/mL (*n* = 3), and high 100 ng/mL (*n* = 3) concentration, and was analyzed for intra-day precision and calculated in the same day, while inter-day precision was calculated via repeated analyses for six consecutive days. The precision and accuracy of the method were established in lower-quality control (LQC), medium-quality control (MQC), and high-quality control (HQC) samples (6 replicates with 3 sets). Percent standard deviation (RSD) is shown in Table 3. The recovery was calculated as the peak area ratio of pre-extraction versus post-extraction spiked samples. For recovery assessment, the above procedure was followed, except analytes were spiked in blank urine before the extraction. Recovery and RSD values were obtained for the quality control samples with low, medium, and high spiking. It was found within a range of 91 ± 7–101 ± 5, as depicted in Table 3.

### 2.7. Extraction Recovery, Matrix Effect, and Carryover

The extraction recoveries and matrix effect were evaluated at three QC levels, i.e., LLOQ (10 ng/mL), QC low (25 ng/mL), and QC high (100 ng/mL), by comparing the peak areas obtained from urine sample with the analytes spiked pre- versus post-extraction spiking in urine matrix. The recovery and matrix effect was calculated at a single point (25 ng/mL) concentration for primary transitions for all three metabolites. Waters Acquity UPLC with strong and weak needle wash was optimized with a partial loop with needle overfill (PLNO) mode used as an injection program to minimize carry-over effects. The sample dilutant solvent used as double blank along with a urine matrix dilution (Single blank) was injected immediately after an injection of the highest concentration of standards to assess carryover.

### 2.8. Data Analysis

MRM signals were digitally captured with acquisition controlling software MassLynx V.4.2 (Waters Corporation, Milford, MA, USA). Quantification of all the metabolites in the urine samples was performed using TargetLynx software V.4.2 (Waters Corporation, Milford, MA, USA) against the calibration curve generated for Daidzein, Geninstein, and S-Equol acid within a range of 0.25 ng–1000 ng/mL. Analyte concentrations were calculated using a 1/x weighted linear regression analysis of the nine-point linear standard curve.

### 2.9. Metabolites Stability

We checked the stability of all the metabolites after the extraction, and describe one of the experiments on precision, accuracy, and recovery with processed samples, in which repeated analyses for all metabolites for six consecutive days were performed at cooling temperature (kept at 4 °C in autosamplers). Results for all three metabolites were observed with intraday variation within ±5 RSD (Table 3). Previous reports also indicated isoflavone stability in urine kept at room temperature for 2 weeks, with no significant degradation of isoflavones reported under these conditions [23].

## 3. Materials and Methods

### 3.1. Chemicals and Reagents

Daidzein, Geninstein, and S-Equol standards were purchased from Target Mol, MA, and LC laboratories, MA, respectively. Acetonitrile (HPLC-grade), Formic acid, MS-grade water, Methanol, and Ammonium acetate were purchased from Sigma Aldrich (St Louis, MO, USA).

### 3.2. Stock Preparation

Stock solutions of Daidzein, Geninstein, and S-Equol were prepared in a 100% Acetonitrile solution; the working stock was in Acetonitrile and Water (80:20). Standard concentrations ranged from 1 to 1000 ng/mL for Daidzein, Geninstein, and S-Equol. Calibration curve standards were prepared in duplicate for absolute quantitation, while QC and blank (non-spiked) samples were prepared in triplicate.

### 3.3. Sample Preparation and Isoflavones Extraction from Urine

Geninstein, S-Equol, and Daidzein absolute quantitation were performed on mouse urine samples. We transferred 50 μL urine samples into 1.5 mL tubes and mixed with 450 μL 10 mM Ammonium acetate (pH 3.7 adjusted with Acetic acid) and incubated at 65 °C for 4 h with 10 min vigorous vortexing and sonication every hour. Samples were placed at room temperature for 10 min followed by a cleaning procedure with strong anion exchange solid phase extraction (SPE). Then, 500 μL of 30% acetonitrile was added to the SPE cartridge. We optimized the SPE-based cleanup procedure for enrichment with all three metabolites and, based on the recovery experiments, the Phenomenex Strata-X PRO (Torrance, CA, USA) 60 mg/3 mL mixed mode strong cation cartridge was found to be most suitable for the development and validation of the final method with 91–101% metabolite recovery. A positive pressure manifold was used to extract all metabolites. SPE was performed using Strata-X PRO, with the following steps: Strata-X was washed with 100% acetonitrile followed by conditioning with 30% acetonitrile before adding urine samples. The 500 μL urine sample was transferred into the already conditioned SPE cartridge. This was followed by washing with 1 mL 50% Acetonitrile solution twice. Finally, 250 μL of Acetonitrile:Methanol (50:50) was added twice to elute metabolites [24]. The final eluent was dried under the N2 evaporator at 40 °C until completely dried. The dried residue was re-suspended in 200 µL diluent (80% Acetonitrile), vortexed, and centrifuged (15 min at 15,000× *g* at 4 °C). The sample was diluted 1:10 with diluent before injection and placed in an autosampler vial for LC-MS/MS analysis.

### 3.4. LC-MS/MS Instrumentation and Conditions

Daidzein, Genistein, and S-Equol working solutions were infused separately to optimize for Q1 and Q3 transitions for their best ionization and fragmentation source parameters (Table 2). A Waters UPLC column connected to Acquity TQD mass spectrometry was employed for method development, including the LC method optimization, ionization, and fragmentation. UPLC was configured with a binary pump, a thermostated column compartment, and a temperature-controlled autosampler. The binary pump was used to transport mobile phase A (2 mm Ammonium acetate +0.2% acetic acid) and B (Acetonitrile +0.2% acetic acid) at a flow rate of 0.30 mL/min in gradient mode. The best separation of Daidzein, Geninstein, and S-Equol was achieved using UPLC with an autosampler with a reversed-phase Waters Acquity CSH (C18, 130 Å, 1.7 µm, 2.1 mm × 150 mm) Column (P/N: 186005298) kept at 65 °C. A 3 min linear gradient employed an initial condition of 60% B with a linear gradient to 80% B at 1.00 min, followed by a linear gradient to 95% B at 0.50 min, followed by a return to 60% B in 0.5 min at 1.0 min and re-equilibration with an initial condition for all isoflavones and isoflavandiol separation. The autosampler was maintained at 4 °C to maintain compound stability and the injection volume was 10 µL with a total running time of 3 min.

TQD mass spectrometry was operated in electrospray ionization mode. The column effluent was monitored using negative ion electrospray (ESI-) using multiple reaction monitoring (MRM) workflows. The primary and confirmatory MRM transitions used for isoflavones and isoflavandiol with their respective optimized settings are listed in Table 2.

## 4. Conclusions

The present study describes a new UPLC-MS/MS methodology coupled with a unique simple extraction method for quantifying total Daidzein, Genistein, and S-Equol in mice urine. It is significantly simpler and quicker than existing published protocols (Table S1) [19,21,24,25,26,27,28]. This is predominantly because this method avoids the laborious extraction procedures, e.g., enzymatic digestion and sulfatases, commonly used for extraction which interfere with the ionization and produce high background noise. The present UPLC-MS/MS method is relatively rapid and sensitive compared to all existing methods for these metabolites and has a high degree of reproducibility in terms of precision, linear range, sensitivity, and repeatability. This method also offers good accuracy without enzymatic digestion for all tested isoflavones; this has never been reported, to the best of our knowledge. The present non-enzymatic and ultra-fast method is suitable for analyzing most dietary intervention studies and investigating relationships between isoflavone consumption and its benefits to human health.

## Figures and Tables

**Table 1:** Method performance parameters for UPLC-MS/MS method including calibration range, regression, LOD, LOQ, S/N, and retention time (P-Primary, C-Confirmatory).

Metabolites	Calibration Range (ng/mL)	r^2^	Limit of Detection (ng/mL)	Limit of Quantitation (ng/mL)	Signal to Noise (s/n)	Retention Time (min)
Daidzein-P	2.0–1000	0.9973	0.5	2.0	23.90	0.69 ± 0.04
Daidzein-C	2.0–1000	0.9964	0.5	2.0	24.45	0.69 ± 0.04
Genistein-P	4.0–1000	0.9973	1.0	4.0	33.84	0.73 ± 0.03
Genistein-C	4.0–1000	0.9899	1.0	4.0	38.56	0.73 ± 0.03
S-Equol-P	2.0–1000	0.9970	0.25	2.0	23.34	0.75 ± 0.05
S-Equol-C	2.0–1000	0.9925	0.25	2.0	29.89	0.75 ± 0.05

**Table 2:** Selected MRM transitions for Daidzein, Geninstein, and S-Equol with the optimized MS parameters for the best sensitivity and selectivity.

Metabolites	Selected MRM Precursor > Fragment	Collision Energy	Cove Voltage	Confirmative Ions	r^2^
Daidzein-P	253.2 > 91	10	32	Primary	0.997
Daidzein-C	253.2 > 223.2	12	32	Confirmatory	0.996
Genistein-P	269 > 133	10	45	Primary	0.997
Genistein-C	269 > 135	12	48	Confirmatory	0.989
S-Equol-P	241.1 > 121.2	12	45	Primary	0.997
S-Equol-C	241.1 > 119.2	10	42	Confirmatory	0.992

**Table 3:** Precision Accuracy with recovery upon spiking all three metabolites at Low, Medium, and High-quality control concentrations along with Mean Intraday and Interday variation in urine.

Metabolites	Low-10 ng/mL Spike		Medium-25 ng/mL Spike		High-100 ng/mL Spike		Intraday Variation	Interday Variation
	Recovery	RSD	Recovery	RSD	Recovery	RSD	RSD	RSD
Daidzein (P)	93 ± 2	5.2	98 ± 6	5.3	96 ± 7	2.1	1.3	4.8
Genistein (P)	96 ± 5	6.1	97 ± 4	6.3	99 ± 3	3.9	2.1	5.0
S-Equol (P)	92 ± 3	5.3	101 ± 5	4.2	91 ± 7	2.3	3.6	4.2

## Data Availability

All data are available within the manuscript.

## Supplementary Materials

The following supporting information can be downloaded at: https://www.mdpi.com/article/10.3390/molecules28186729/s1. Table S1: Summary of few previously reported Isoflavone methods with newly developed method with same Matrix (Urine) and LOQ.

## Sample Availability

Samples of the compounds are available from the authors.
