# Supplementary material for: A UPLC-MS/MS Based Rapid, Sensitive, and Non-Enzymatic Methodology for Quantitation of Dietary Isoflavones in Biological Fluids
Source: Molecules. 2023 Sep 21;28(18):6729. doi: 10.3390/molecules28186729 (PMC10534480; doi:10.3390/molecules28186729)
Supplement: Supplementary file 1 [file molecules-28-06729-s001.zip › molecules-2579728-supplementary.pdf]

Supplementary Table **S1**

Summary of few previously reported Isoflavone methods with newly developed method with same Matrix (Urine) and LOQ may be added as supplement information if needed.

Author	Enzyme based Extraction	Matrix (Urine)	Analysis Time	Diadzein (ng/ml)	Genistein (ng/ml)	S-equol (ng/ml)
Landon Wilson (2014)	β-glucuronidase /sulfatase	Urine	30 mins	1.2	0.15	1.27
Yaoyue Liang (2018)	no enzyme	Urine	30 mins	157	157	157
Soukup (2014)	no enzyme	Urine/Plasma	25 mins	6.4	3.8	63.5
Philip B. Grace (2007)	β-glucuronidase /sulfatase	Urine/Plasma	10 mins	2.0	2.0	2.0
Current Method	No enzyme	urine	05 mins	2.2	4.0	2.0

25. Wilson L, Arabshahi A, Simons B, Prasain JK, Barnes S. Improved high sensitivity analysis of polyphenols and their metabolites by nano-liquid chromatography-mass spectrometry. Arch Biochem Biophys. 2014 Oct 1;559:3-11. doi: 10.1016/j.abb.2014.06.014. Epub 2014 Jun 23. PMID: 24967696; PMCID: PMC4143533.

26. Liang Y, Zhao W, Wang C, Wang Z, Wang Z, Zhang J. A Comprehensive Screening and Identification of Genistin Metabolites in Rats Based on Multiple Metabolite Templates Combined with UHPLC-HRMS Analysis. Molecules. 2018 Jul 26;23(8):1862. doi: 10.3390/molecules23081862. PMID: 30049985; PMCID: PMC6222673.

27. Soukup, S. T., Al-Maharik, N., Botting, N., & Kulling, S. E. (2014). Quantification of soy isoflavones and their conjugative metabolites in plasma and urine: an automated and validated UHPLC-MS/MS method for use in large-scale studies. Analytical and Bioanalytical Chemistry, 406(24), 6007–6020. doi:10.1007/s00216-014-8034-y.

28. Philip B. Grace, Nayan S. Mistry, Megan H. Carter, Anthony J.C. Leathem, Philip Teale, High throughput quantification of phytoestrogens in human urine and serum using liquid chromatography/tandem mass spectrometry (LC–MS/MS),Journal of Chromatography B,Volume 853, Issues 1–2,2007,Pages 138-146.
